# Differences between lung adenocarcinoma and squamous cell carcinoma in histological distribution of residual tumor after induction chemoradiotherapy

**DOI:** 10.1007/s12672-021-00431-8

**Published:** 2021-09-27

**Authors:** Hiroaki Nomori, Atsushi Shiraishi, Koichi Honma, Kazufusa Shoji, Ayumu Otsuki, Yue Cong, Hiroshi Sugimura, Yu Oyama

**Affiliations:** 1 Department of Thoracic Surgery, Kashiwa Kousei General Hospital, 617 Shikoda, Kashiwa City, Chiba, 277-8661 Japan; 2 Department of Emergency and Trauma Center, Kameda Medical Center, Chiba, Japan; 3 Department of Pathology, Kameda Medical Center, Chiba, Japan; 4 Department of Radiology, Kameda Medical Center, Chiba, Japan; 5 Department of Thoracic Pulmonary Medicine, Kameda Medical Center, Chiba, Japan; 6 Department of Thoracic Surgery, Kameda Medical Center, Chiba, Japan; 7 Department of Medical Oncology, Kameda Medical Center, Chiba, Japan; 8 Department of Minimally Invasive Surgical and Medical Oncology, Fukushima Medical University, Fukushima, Japan

**Keywords:** Lung cancer, Adenocarcinoma, Squamous cell carcinoma, Induction chemoradiotherapy, Tumor response, Radiosensitivity

## Abstract

**Aims:**

To facilitate dose planning for convergent beam radiotherapy in non-small cell lung cancer (NSCLC), tumor response and histological distribution of residual tumors after induction chemoradiotherapy (ICRT) were compared between adenocarcinoma (AD) and squamous cell carcinoma (SQ).

**Methods:**

Ninety-five patients with N1–2 or T3–4 NSCLC were treated with ICRT followed by surgery; 55 had AD and 40 had SQ. For the evaluation of distribution of residual tumors, the location of the external margin of residual tumors was assessed on surgical materials as follows: radius of whole tumor (“a”); distance between the center of tumor and the external margin of residual tumor (“b”); and its location (“b/a”).

**Results:**

Of the 55 AD cases, 8 (15%) showed pathological complete remission, which was significantly less frequent than 22 of 40 SQ cases (55%) (p < 0.001). AD showed the residual tumors at the most periphery of tumor (b/a = 1.0) more frequently than SQ, i.e., 39/55 (71%) versus 6/40 (15%), respectively (p < 0.001). Even in 65 cases other than the pathological complete remission, external margins in 47 AD cases located more periphery than those in 18 SQ cases, of which mean b/a values were 0.97 ± 0.17 and 0.70 ± 0.29, respectively (p < 0.001).

**Conclusion:**

AD showed worse tumor response to ICRT than SQ. After ICRT, AD remained at the periphery of primary tumor more frequently than SQ. It seems that, also in the convergent beam radiotherapy, the periphery part of AD would be more resistant than that of SQ.

**Supplementary Information:**

The online version contains supplementary material available at 10.1007/s12672-021-00431-8.

## Introduction

Radiotherapy is an important treatment modality for lung cancer, of which ultimate goal is to achieve local tumor control while sparing the surrounding normal tissue to limit toxicities. While a stereotactic radiotherapy has been used as a convergent beam therapy for non-small cell lung cancer (NSCLC), an intensity-modulated radiotherapy (IMRT) or volumetric modulated arc therapy (VMAT) is recently used to adjust a radiation dose distribution (i.e., dose painting) around the tumor to decrease damage of surrounding normal tissue [1, 2]. However, the IMRT and VMAT adjust the radiation dose only around tumor but not within it, because the distribution of radiosensitivity within a tumor has not been clarified, which could cause local recurrence.

To clarify the distribution of radiosensitivity within a tumor, a pathological examination of tumors treated by radiotherapy is necessary, however preoperative radiotherapy is rarely conducted these days. While fluorodeoxyglucose (FDG) uptake on positron emission tomography (PET) would be a way to predict the residual tumor within the tumor after radiotherapy, FDG-uptake might decrease early thereafter due to the temporary weakening of tumor cells but could increase again later [3]. Therefore, the elucidation of the distribution of radiosensitivity within a primary site of NSCLC currently depends on examination of the distribution of residual tumors on surgical materials after induction chemoradiotherapy (ICRT) [4–8], which has been frequently used for locally advanced NSCLC. While two studies evaluated the difference in tumor responses after ICRT between adenocarcinoma (AD) and squamous cell carcinoma (SQ) [9, 10], no reports have examined the differences in residual tumor locations after ICRT.

Therefore, the present study examined the difference between AD and SQ in tumor response and histological distribution of residual tumors after ICRT. It also examined the differences between the two in recurrence-free survival (RFS) and overall survival (OS).

## Methods

### Study design

The present study was a single-center retrospective and observational study. The study design adhered to the Strengthening the Reporting of Observational Studies in Epidemiology guidelines [11]. Based on the guidelines for lung cancer published by the Japanese Lung Cancer Society [12], we established a protocol for ICRT followed by surgery for patients with locally advanced NSCLC in October 2012, that was adopted by the Lung Cancer Board of Kameda Medical Center, an educational and cancer-designated hospital that surgically treats more than 120 patients with lung cancer annually. The retrospective analysis protocol for patients treated with ICRT followed by surgery was approved by the institutional ethics committee in 2014 (approval number: 14–005). PET/computed tomography (PET/CT) was conducted before and after ICRT to determine the final indications of surgery. All patients provided informed consent after the attending physicians explained the risks and benefits of ICRT followed by surgery.

### Eligibility

The study participants of the present study fulfilled the following criteria: (1) N2 stage diagnosed via endobronchial ultrasound-guided transbronchial needle aspiration or FDG-PET; (2) N1 stage disease with a locally invasive tumor; (3) T3 or T4 stage disease diagnosed via CT and magnetic resonance imaging; (4) prediction of tolerance to ICRT followed by surgery; and (5) patients preferred ICRT followed by surgery over definitive chemoradiotherapy. Tumor staging was based on the eighth edition of the TNM Classification proposed by the International Association for Study of Lung Cancer [13].

### Data source

Between October 2012 and December 2018, a total of 107 patients with locally advanced NSCLC were treated with ICRT at Kameda Medical Center. Comorbidity was assessed using the Charlson comorbidity index [14]. ICRT was administered as a concurrent chemoradiotherapy regimen using a platinum doublet agent.

### Clinical response evaluation

Clinical response was evaluated on CT according to the Response Evaluation Criteria in Solid Tumors criteria [15]. Change in the tumor size was measured as the ratio of the tumor size after versus before ICRT.

### Pathological response

Pathological findings on surgical materials were reviewed by a pathologist (K.H.). The pathological response was defined according to the criteria given in the “General Rules of Clinical and Pathological Records of Lung Cancer in Japan” as follows [16]: Ef.0 was defined as no therapeutic response; Ef.1a was defined as a viable tumor greater than or equal to two-thirds of the tumor; Ef.1b was defined as a viable tumor greater than or equal to one-third of the tumor and less than two-thirds; Ef.2 was defined as a viable tumor less than one-third of the tumor; and Ef.3 was defined as no viable tumor cells, i.e. pathological complete remission (CR).

### Measurement of the location of external margins of residual tumors

**Fig. 1:**
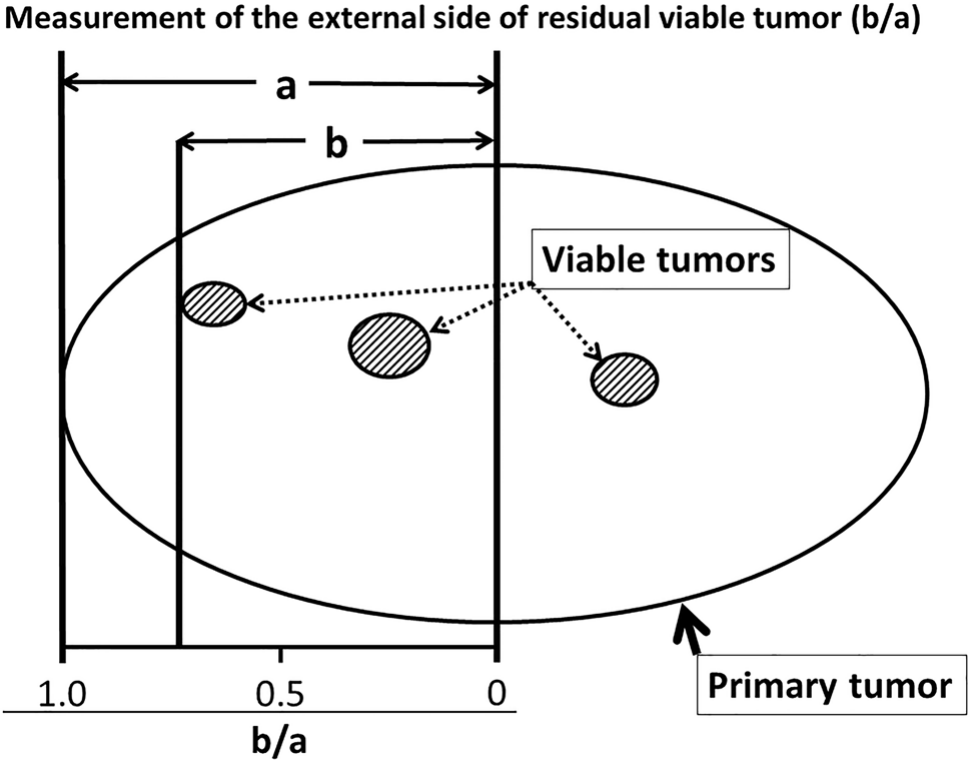
Measurement of the external margin of the residual tumor (b/a); “a” is the radius of the whole tumor, while “b” is the distance between the center of the tumor and the external margin of the residual tumor

Figure 1 shows a method for measuring the location of the external margins of the residual tumors within the primary tumor. Residual tumors were mapped on the grossly cut surface by examination of the hematoxylin–eosin-stained sections of the surgical materials. The location of the external margin of the residual tumor was measured as follows: (1) the radius of the whole tumor (“a”) and the distance between the center of the tumor and the external margin of the residual tumor (“b”); and (2) the location of the external margin of the residual tumor was determined as “b/a”. The external margins of residual tumors were determined by 2 authors, i.e., a surgeon (H.N.) and a pathologist (K.H.).

### Analysis of FDG-PET data

A PET/CT device (Discovery ST; GE Medical Systems, Amersham, UK) was used to perform FDG-PET scanning before and after ICRT. FDG uptake of the primary tumor was measured using the standardized uptake value (SUV). Change in the SUV was measured as the ratio of the SUV after versus before ICRT.

### Follow-up

Postoperative follow-up was conducted by body CT and brain magnetic resonance imaging every 3 or 4 months until 3 years after surgery and a minimum of every 6 months thereafter. Follow-up data were collected from the medical records in June 2020.

### Study outcomes

The primary outcome was the differences in the tumor response and the location of the external margin of the residual tumor between AD and SQ groups. The secondary outcomes included the differences in RFS and OS between the two groups.

### Statistical analysis

Difference in the “b/a” between AD and SQ was analyzed by using the Mann–Whitney U-test. Changes in tumor size and SUV value after ICRT in each patient were analyzed using the paired Wilcoxon signed-rank test. Differences between AD and SQ in the tumor size and SUV before ICRT and in the changes of tumor size and SUV after ICRT were analyzed by using the Mann–Whitney U-test. Differences between AD and SQ in nominal variables were analyzed using the χ^2^ test. The RFS and OS after ICRT were assessed using the Kaplan–Meier method [17]. All values in the text and tables are presented as the means ± standard deviation. Statistical significance was set at P-values of < 0.05. The statistical analyses were performed using Microsoft Excel for Windows 10.

## Results

**Fig. 2:**
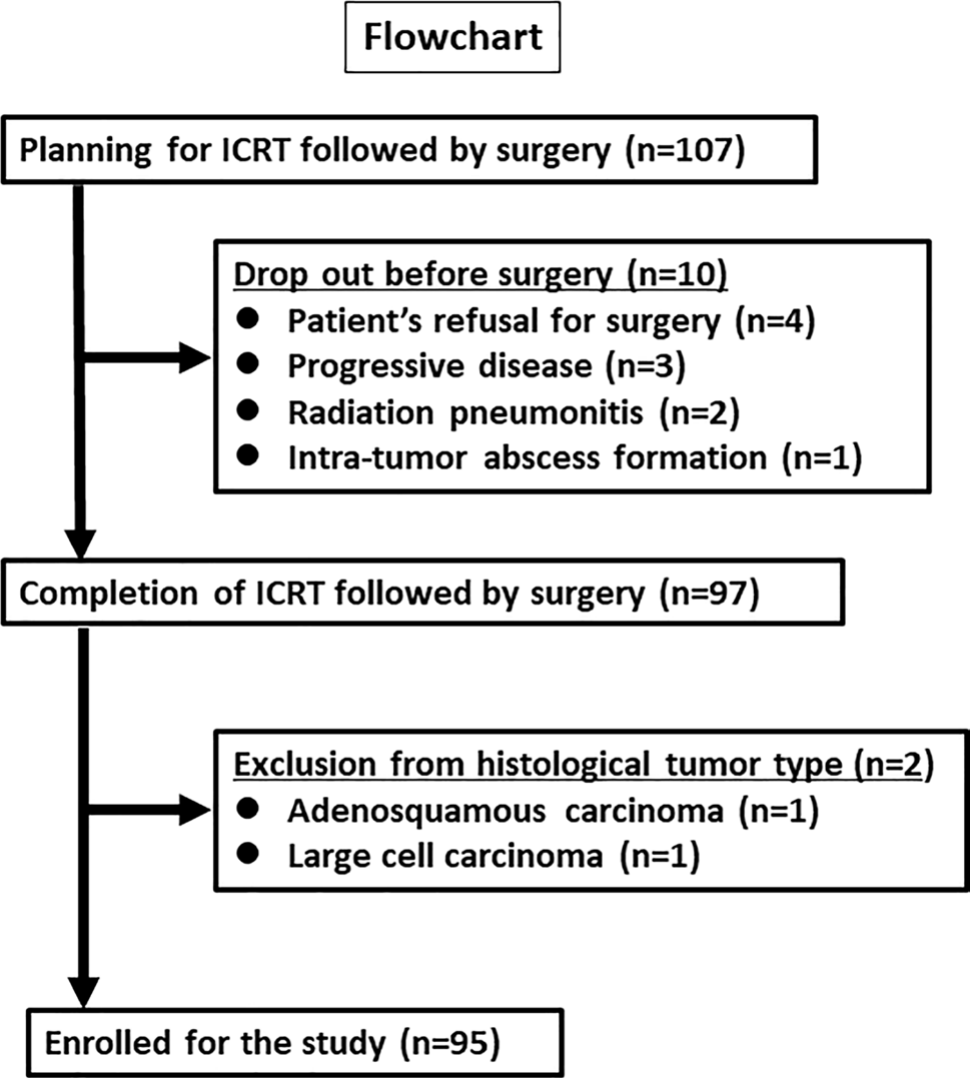
Flowchart of the patient selection process

Figure 2 shows a flowchart showing the patient selection process. Of the 107 patients treated with ICRT, surgery was not performed in 10 patients. Two patients were excluded for having tumor types other than AD or SQ. Finally, 95 patients were enrolled in the study. Mean number of chemotherapy cycles for the ICRT was 2.1 ± 0.7. Chemotherapy regimens were carboplatin and paclitaxel in 46 patients, cisplatin and docetaxel in 46, and cisplatin and pemetrexed in 3. The radiation dose was 40 Gy for 49 patients, 46 Gy for 42, 50 Gy for 2, and 60 Gy for 2.

Table 1 shows the characteristics of the patients with AD (n = 55) versus those with SQ (n = 40) before ICRT. SQ showed a larger tumor size (5.8 ± 1.9 vs. 4.6 ± 1.9 cm) and higher SUV (14.6 ± 6.1 vs. 10.7 ± 6.0) than AD (p = 0.002 and 0.006, respectively). There were no significant differences in sex, age, comorbidity index, tumor location, clinical stage, number of chemotherapy cycles, radiation dose (40 Gy vs. ≥ 46 Gy), and surgical procedures between AD and SQ (p = 0.86, 0.62, 0.10, 0.07, 0.46, 0.32, 0.57, and 0.12, respectively).

**Table 1:** Patient characteristics in adenocarcinoma and squamous cell carcinoma

Total (%)	Adenocarcinoma	Squamous cell carcinoma	Difference
	55 (100)	40 (100)	
Patient			
Sex = male (%)	46 (84)	34 (85)	p = 0.86
Age (years old)	63 ± 10	67 ± 9	p = 0.62
Comorbidity index	2.6 ± 0.6	2.8 ± 0.5	p = 0.10
Tumor			
Tumor size (cm)	4.6 ± 1.9	5.8 ± 1.9	p = 0.002
SUV	10.7 ± 6.0	14.6 ± 6.1	p = 0.006
Central location	8 (15)	12 (30)	p = 0.07
Clinical stage			
IIB	16 (29)	6 (15)	p = 0.46
IIIA	19 (35)	17 (43)	
IIIB	16 (29)	17 (43)	
IIIC	4 (7)	0 (0)	
Treatment			
Number of chemotherapy cycles			
1	4 (7)	2 (5)	p = 0.32
2	46 (84)	37 (93)	
≥ 3	5 (9)	1 (3)	
Radiation dose			
40 Gy	27 (49)	22 (55)	p = 0.57
≥ 46 Gy	28 (51)	18 (45)	
Surgical procedures			
Segmentectomy	5 (9)	0 (0)	p = 0.12
Lobectomy	49 (89)	39 (98)	
Pneumonectomy	1 (2)	1 (3)	

*SUV* standard uptake value on positron emission tomography

Central location: tumors presented at the central to segmental bronchus

Table 2 shows the clinical TNM stages before ICRT, which did not show a significant difference of N0 stage between the groups, i.e., 11 of 55 AD patients (20%) and 3 of 40 SQ patients (8%) (p = 0.14). There was also no significant difference of T3/T4 stages between the groups, i.e., 32 of 55 patients (58%) in AD and 30 of 40 (75%) in SQ (p = 0.12).

**Table 2:** Clinical TNM stage before induction chemoradiotherapy in adenocarcinoma and squamous cell carcinoma

Total	Adenocarcinoma	Squamous cell carcinoma
	55	40
Clinical TNM		
T1N1M0	1	1
T1N2M0	7	2
T1N3M0	1	0
T2N1M0	6	3
T2N2M0	7	4
T2N3M0	1	0
T3N0M0	9	2
T3N1M0	3	10
T3N2M0	8	3
T3N3M0	4	0
T4N0M0	2	1
T4N2M0	6	14

Difference in N0-stage cases was not different between adenocarcinoma and squamous cell carcinoma (p = 0.14). Difference in T3/T4 cases was not significant difference between the two (p = 0.12)

**Fig. 3:**
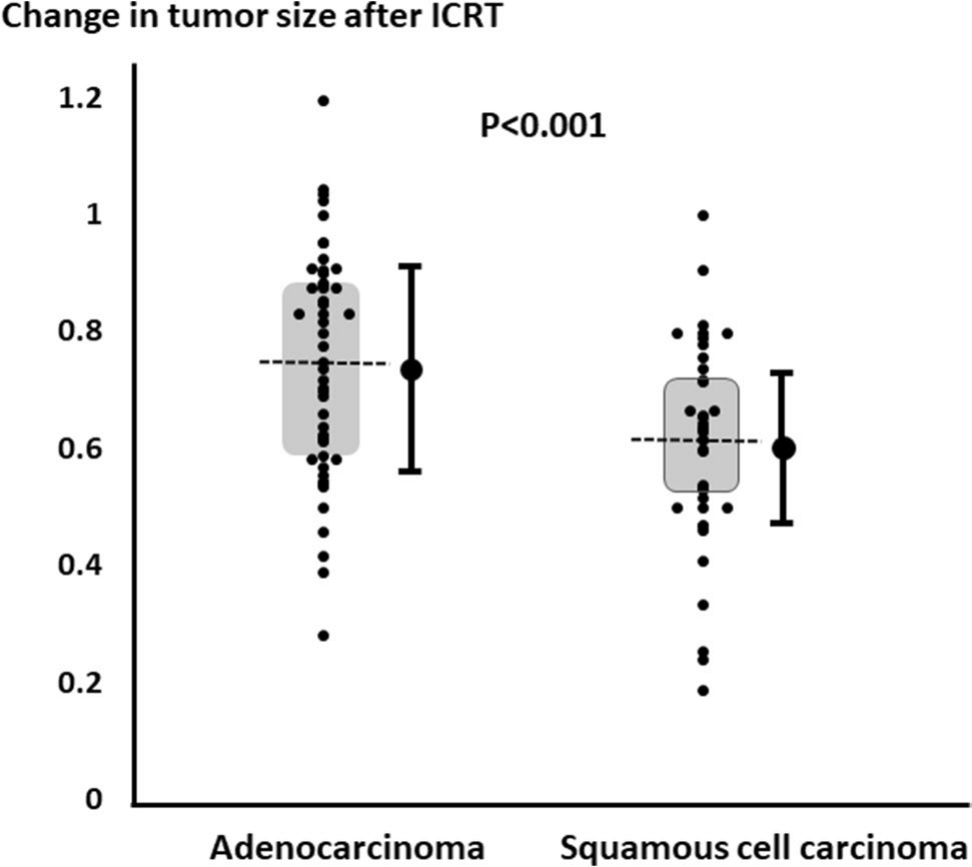
Changes in tumor size after induction chemoradiotherapy for adenocarcinoma and squamous cell carcinoma. Squamous cell carcinoma showed significantly greater reductions in tumor size than adenocarcinoma (p < 0.001). Shadow area showed the first quartile and the third quartile. The dotted line showed the median value

The tumor sizes of AD and SQ groups were significantly reduced after ICRT (p < 0.001), i.e., from 4.6 ± 1.9 cm before ICRT to 3.2 ± 1.6 cm after ICRT for AD, and from 5.8 ± 1.9 cm before ICRT to 3.4 ± 1.5 cm after ICRT for SQ. Figure 3 shows the change ratio in tumor size after ICRT, which was 0.74 ± 0.19 for AD and 0.61 ± 0.18 for SQ; SQ showed a significant decrease in tumor size compared to AD (p < 0.001). AD showed a partial response less frequently than SQ with significance (p = 0.016), i.e., 25/55 (46%) and 29/40 (73%), respectively.

All patients underwent PET before and after ICRT. The SUV of both AD and SQ significantly reduced after ICRT (p < 0.001), i.e., from 10.7 ± 6.0 before ICRT to 5.2 ± 4.0 after ICRT for AD, and from 14.6 ± 6.1 before ICRT to 4.2 ± 3.5 after ICRT for SQ. The change ratio of SUV after ICRT was significantly lower in the SQ group (0.32 ± 0.28) than in the AD group (0.53 ± 0.27) (p < 0.001) (Figure S1).

Lobectomy was performed in 88 patients, segmentectomy in 5, and pneumonectomy in 2. For all 4 cases with N3 stage before ICRT, the N3 stations were dissected; no metastases were revealed. Of the 88 patients who underwent lobectomy, 18 (20%) required bronchial reconstruction. Fifty-one of the 95 patients (54%) received the combined resection for T3/T4 disease. While 91 patients (96%) underwent complete resection (R0), the remaining 4 could not (R1 in 2 and R2 in 2) due to tumor remnants in the aorta, right main bronchus, vertebra, and esophagus, respectively. While 93 patients were discharged without major complications, the other 2 patients died of surgery-related complications.

The pathological tumor responses in AD were Ef.0–1 in 16 patients (29%), Ef.2 in 31 (56%), and Ef.3 (CR) in 8 (14%), and in SQ, the responses were 3 (8%), 15 (37%), and 22 (55%), respectively (Table 3). The SQ showed Ef.3 more frequently than AD (p < 0.001). However, the pathological N0 stage was not significantly different between AD and SQ, i.e., 33 of 55 (60%) and 32/40 (90%), respectively (p = 0.07) (Table S1).

**Table 3:** Histological tumor types and pathological response

Pathological response	Histological type		Total
	Adenocarcinoma	Squamous cell carcinoma	
Ef 0–1	16	3	19
Ef 2	31	15	46
Ef 3	8	22	30
Total	55	40	95

Difference in Ef.3 (complete remission) between adenocarcinoma and squamous cell carcinoma is significant (p < 0.001)

**Fig. 4:**
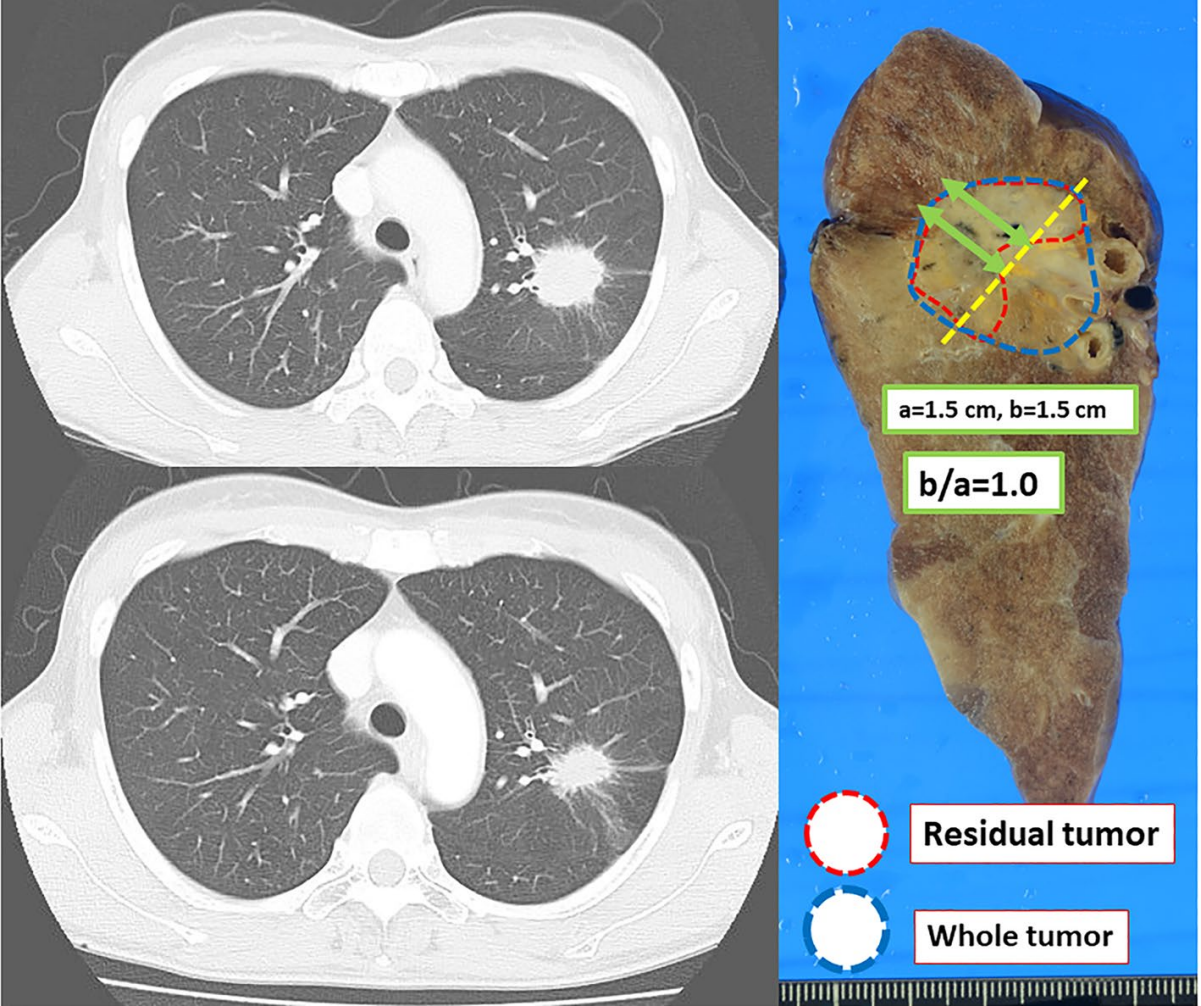
CT images before and after ICRT and the residual tumor location on surgical materials in adenocarcinoma. Residual tumor was located at the periphery side of primary tumor, of which b/a was 1.0

**Fig. 5:**
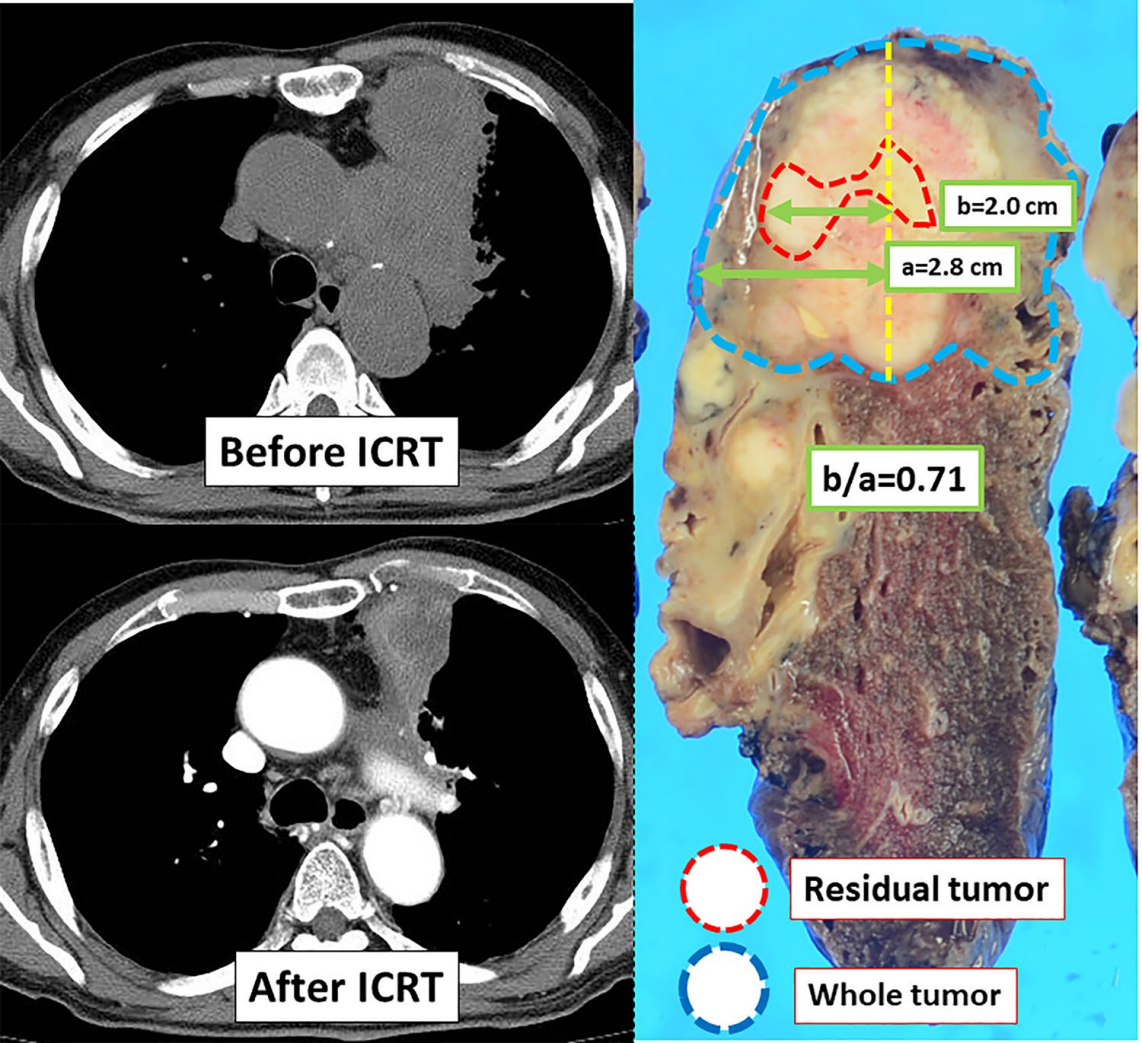
CT images before and after ICRT and the residual tumor location on surgical materials in squamous cell carcinoma. Residual tumor was located in a part near the center of primary tumor, of which b/a was 0.71

**Fig. 6:**
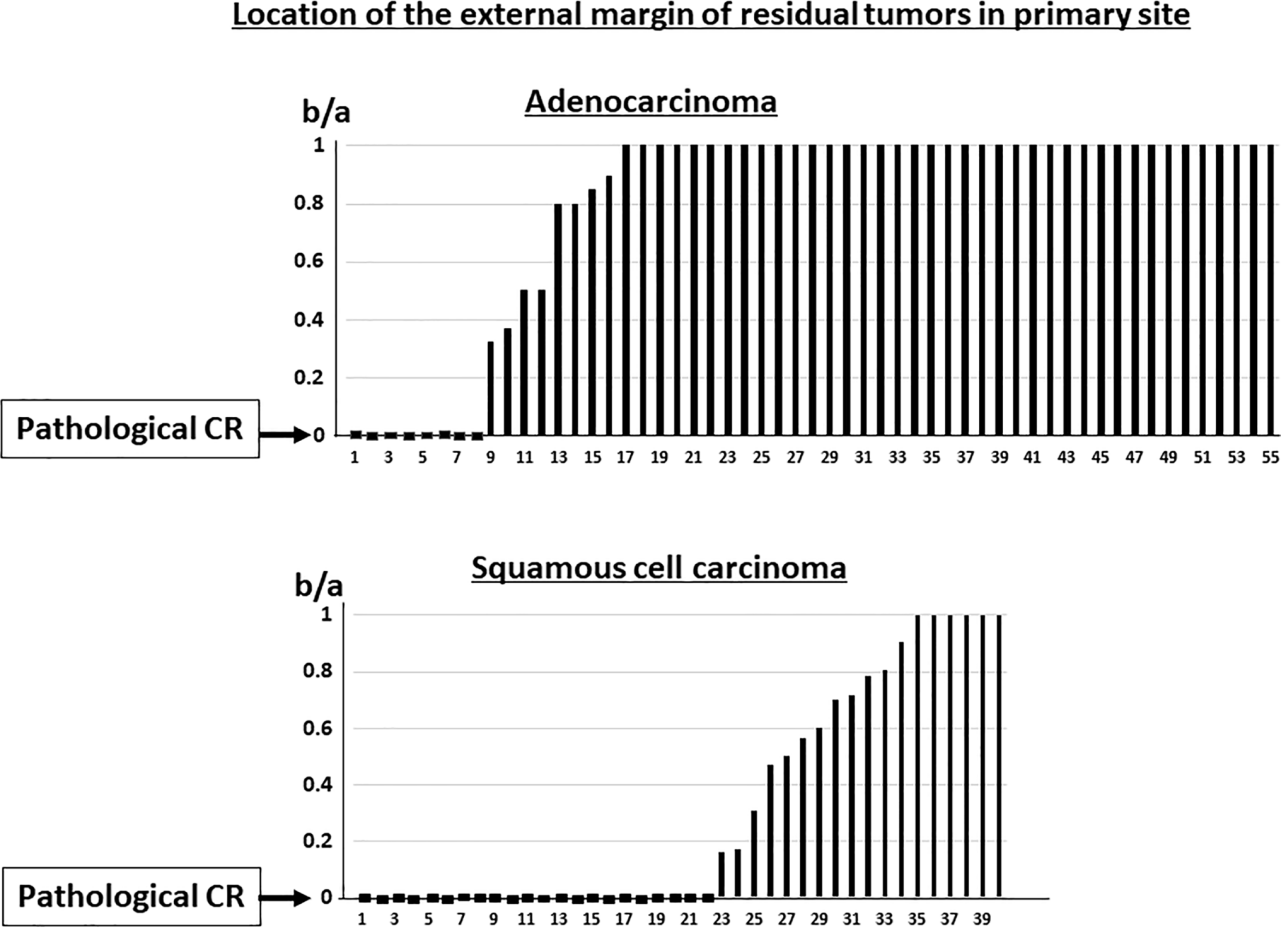
Waterfall plots of the locations of the external margins of the residual tumor (b/a). Thirty-nine of 55 adenocarcinomas (71%) showed the residual tumors at the periphery of the primary tumor (b/a = 1.0), which was significantly frequently than 6 of 40 squamous cell carcinomas (15%) (p < 0.001). Pathological CR: pathological complete remission

External margins of residual tumors in AD were frequently seen at the periphery of tumor than those in SQ (Figs. 4 and 5). Figure 6 shows waterfall plots of the locations of the external margins of residual tumors (b/a). Of the 55 AD cases, 39 (71%) showed residual tumors at the periphery of the primary tumor (b/a = 1.0) in contrast to only 6 of 40 SQ cases (15%); the difference was significant (p < 0.001). The mean b/a value was 0.80 ± 0.37 in AD (median value, 1.0; interquartile range [IQR], 0.18), which was significantly higher than 0.33 ± 0.41 in SQ (median, 0; IQR, 0.71) (p < 0.001). Even in the 65 cases other than the pathological CR, AD still showed residual tumors at the periphery of the primary tumor (b/a = 1.0) more frequently than SQ (p < 0.001), i.e., 39 of the 47 (83%) and 6 of 18 (33%), respectively. The mean b/a value in the 65 patients other than pathological CR was 0.97 ± 0.17 (median value, 1.0; IQR, 0) in AD, which was significantly higher than 0.70 ± 0.29 (median value, 0.75; IQR, 0.48) in SQ (p < 0.001).

None of the patients were lost to follow-up. The median follow-up period was 35 months (range: 4–76 months). Forty-two patients (44%) received adjuvant postoperative chemotherapy: 26 of 55 AD patients (47%) and 16 of 40 SQ patients (40%), of which difference was not significant (p = 0.48). During the study period, 40 patients experienced recurrence (31 with AD, 9 with SQ) and 25 patients died (13 with AD, 12 with SQ). Among the 25 patients who died, 18 died of lung cancer and the other 7 died of other diseases, including surgery-related death in 2 patients.

**Fig. 7:**
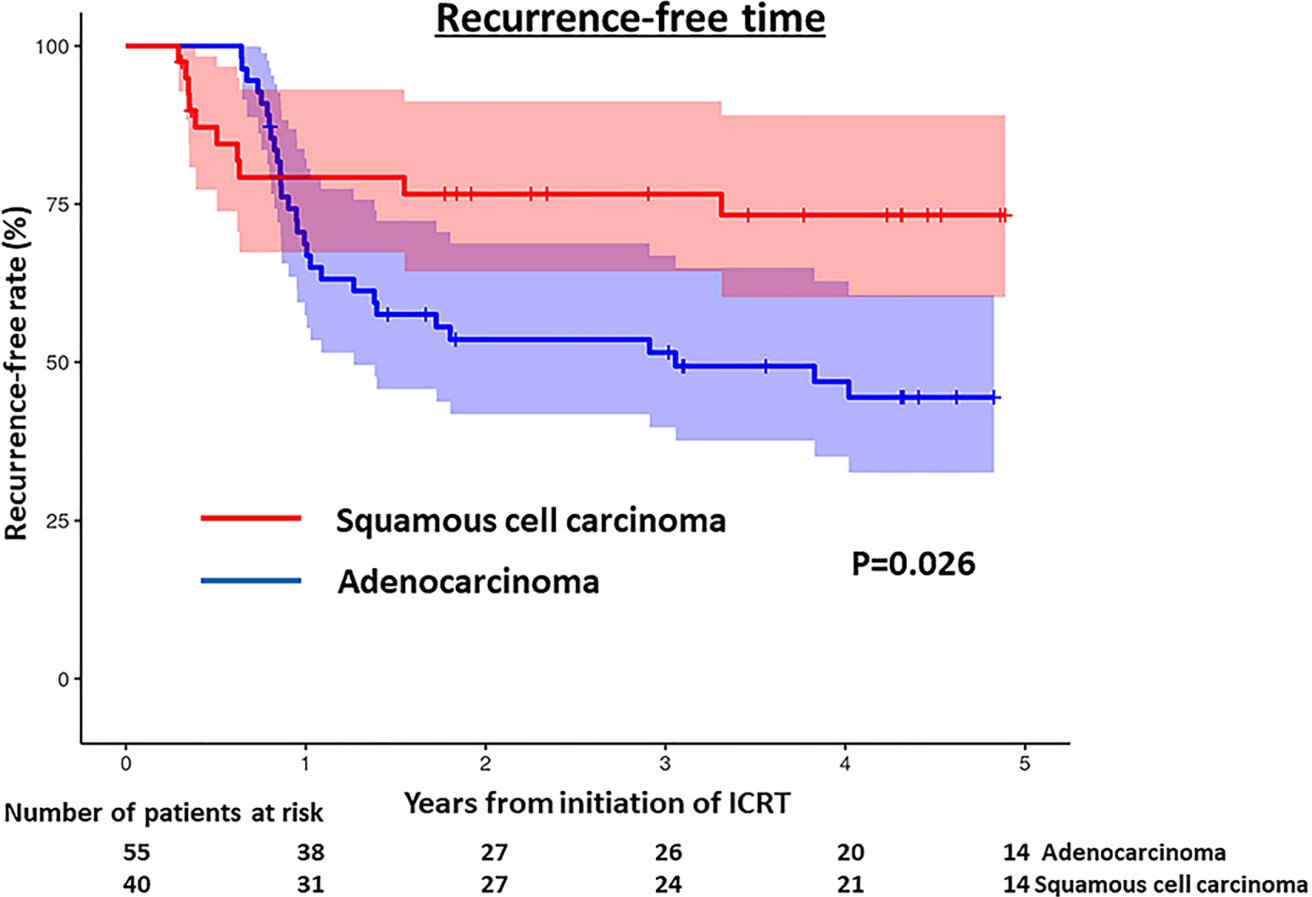
Recurrence-free survival of patients with adenocarcinoma and those with squamous cell carcinoma. Recurrence-free survival was better in patients with squamous cell carcinoma than in those with adenocarcinoma (p = 0.023). The 95% confidence intervals are shown as colored shade area (blue: adenocarcinoma; red: squamous cell carcinoma)

Figure 7 shows the RFS, which was significantly worse in patients with AD than in those with SQ (p = 0.023, log-rank test), with 3-years RFS rates of 45% and 72%, respectively. For the 40 patients with recurrence, additional chemotherapy was administered to 26 of 31 patients with AD (84%) and 7 of 9 patients with SQ (78%), of which difference was not significant (p = 0.62).

**Fig. 8:**
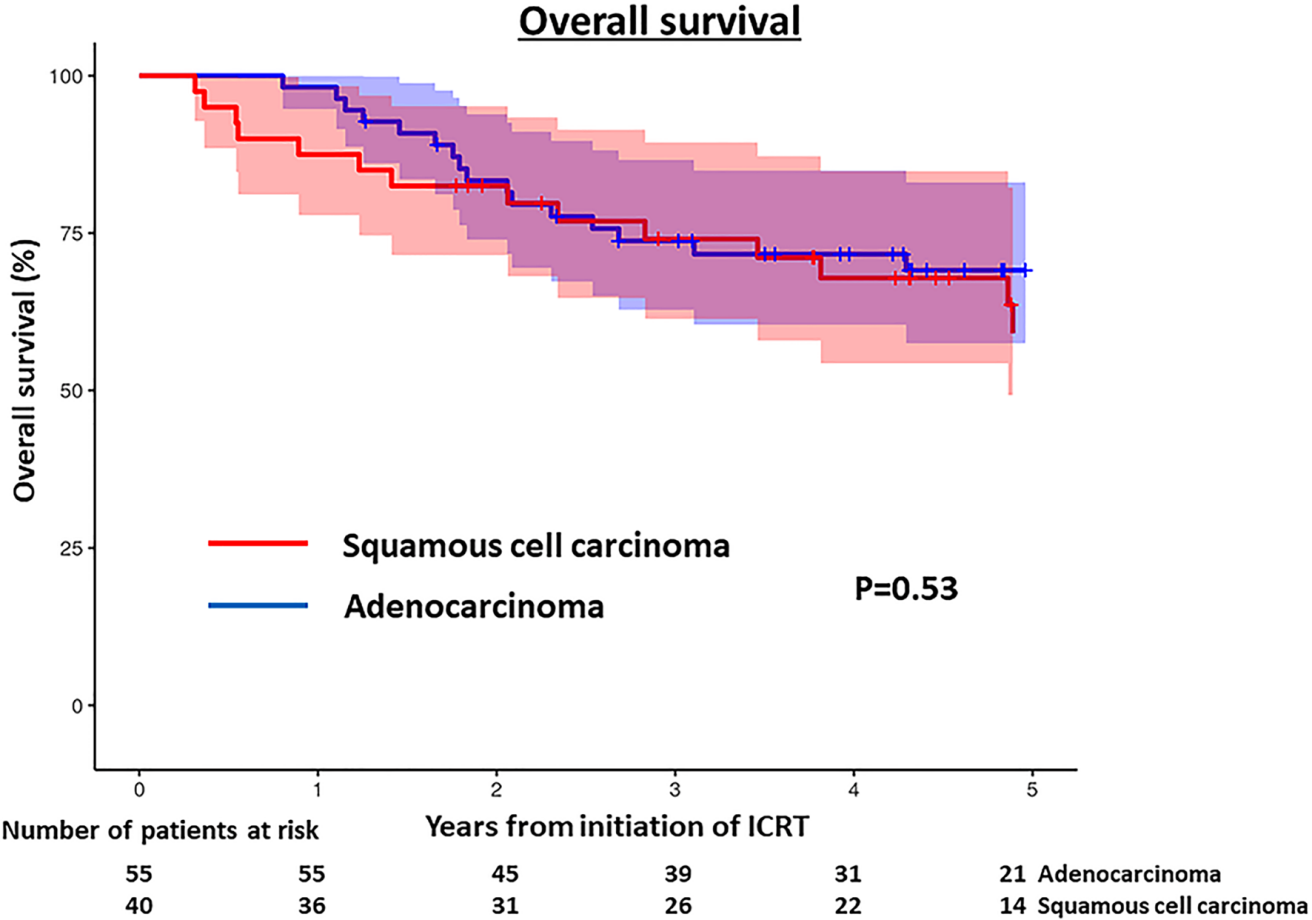
Overall survival of patients with adenocarcinoma and those with squamous cell carcinoma. Overall survival was not significantly different between the two (p = 0.45). The 95% confidence intervals are shown as colored shade area (blue: adenocarcinoma; red: squamous cell carcinoma)

Figure 8 shows the OS, which was not significantly different between AD and SQ (p = 0.45, log-rank test), with 3-years survival rates of 76% and 75%, respectively.

## Discussion

The present study clarified the following points: (1) AD showed the residual tumor at the periphery of the primary tumor after ICRT more frequently than SQ; (2) AD showed worse clinical and pathological responses after ICRT than SQ; and (3) RFS was significantly worse in AD patients than in SQ patients, but there was no significant difference in OS between the two.

No previous studies evaluated the histological locations of residual tumors after ICRT in NSCLC. The present study showed that the AD remained at the periphery of the primary tumor after ICRT more frequently than SQ, suggesting that the periphery part of AD tumors is more resistant to ICRT than that of SQ tumors. The radio-sensitivity of tumors is reportedly dependent on intracellular oxygen concentration [18, 19]. It is well known that the periphery of AD frequently consists of well-differentiated tumor [20, 21], which has less vascularity than SQ. Therefore, the periphery of AD may have a lower sensitivity to radiation therapy. IMRT or VMAT is recently used to adjust a radiation dose distribution (i.e., dose painting) around the tumor to decrease damage of surrounding normal tissue [1, 2]. However, both IMRT and VMAT do not adjust the radiation dose within the primary tumor, because the radiosensitivity distribution within a tumor has not been clarified. The present study showed that AD frequently remained at the periphery of the primary tumor, while SQ did not; this finding could help the determination of dose distribution within a primary tumor of NSCLC. In the dose planning of IMRT or VMAT, the radiation dose around the tumor could be saved in SQ, but not in AD. On the other hand, SQ is well known to have necrosis or cavity in the central part of tumors, suggesting less vascularity there. Therefore, for curative radiation for large SQ tumors, an increased radiation dose near the central part of primary tumor would be necessary.

While RFS was better in patients with SQ than in patients with AD, there was no significant difference in OS regardless of the similar additional chemotherapy administered after recurrence, which could be due to the following reasons: (1) AD would have lower tumor aggressiveness than SQ, resulting in longer survival after recurrence; and (2) AD might be more sensitive to chemotherapy, such as molecular targeted therapy, than SQ.

The present study had some limitations. While the present study aimed to aid dose planning for IMRT or VMAT for lung cancer, the patients were treated by chemoradiotherapy, but not only by radiotherapy; thus, the results could be influenced by chemotherapy. In addition, the chemotherapy regimens and the radiation dose in the ICRT were not standardized.

We concluded that AD was usually more resistant to ICRT than SQ and remained at the periphery of the primary tumor more frequently than SQ. In the IMRT or VMAT, AD would need more radiation dose than SQ for cure, especially at the periphery side of the tumor.

## Supplementary Information

Supplementary file 1. Figure S1. Changes in standardized uptake value in fluorodeoxyglucose positron emission tomography after induction chemoradiotherapy for adenocarcinoma and squamous cell carcinoma. Squamous cell carcinoma showed significantly greater reductions in standardized uptake value than adenocarcinoma (p < 0.001). Shadow area showed the first quartile and the third quartile. The dotted line showed the median value. SUV: standardized uptake value.

Supplementary file 2. Table S1. Pathological TNM stage in adenocarcinoma and squamous cell carcinoma.
