# Supplementary figures and images for: Differences between lung adenocarcinoma and squamous cell carcinoma in histological distribution of residual tumor after induction chemoradiotherapy
Source: Discov Oncol. 2021 Sep 27;12:36. doi: 10.1007/s12672-021-00431-8 (PMC8777543; doi:10.1007/s12672-021-00431-8)

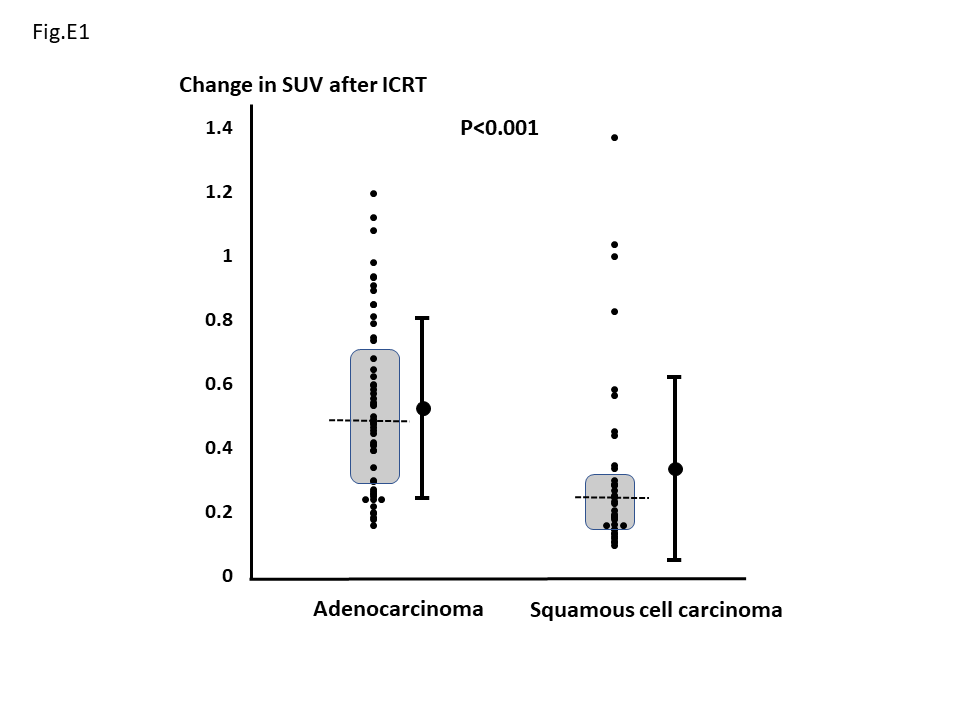

Supplement: Supplementary file 1 — Figure S1. Changes in standardized uptake value in fluorodeoxyglucose positron emission tomography after induction chemoradiotherapy for adenocarcinoma and squamous cell carcinoma. Squamous cell carcinoma showed significantly greater reductions in standardized uptake value than adenocarcinoma (p < 0.001). Shadow area showed the first quartile and the third quartile. The dotted line showed the median value. SUV: standardized uptake value. [file 12672_2021_431_MOESM1_ESM.tif]
