# Supplementary material for: Differences between lung adenocarcinoma and squamous cell carcinoma in histological distribution of residual tumor after induction chemoradiotherapy
Source: Discov Oncol. 2021 Sep 27;12:36. doi: 10.1007/s12672-021-00431-8 (PMC8777543; doi:10.1007/s12672-021-00431-8)
Supplement: Supplementary file 2 — Table S1. Pathological TNM stage in adenocarcinoma and squamous cell carcinoma. [file 12672_2021_431_MOESM2_ESM.docx]

Table S1. Pathological TNM stage in adenocarcinoma and squamous cell carcinoma.

Adenocarcinoma Squamous cell carcinoma

Total 55 40

Pathological TNM

T1N0M0 7 6

T1N1M0 2 0

T1N2M0 8 1

T2N0M0 8 7

T2N1M0 1 2

T2N2M0 5 0

T3N0M0 16 8

T3N1M0 1 1

T3N2M0 3 2

T4N0M0 2 10

T4N1M0 1 0

T4N2M0 1 3

Difference in pathological N0-stage cases was not different between adenocarcinoma and squamous cell carcinoma (p=0.07).
